# Universal Stem-Loop Primer Method for Screening and Quantification of MicroRNA

**DOI:** 10.1371/journal.pone.0115293

**Published:** 2014-12-30

**Authors:** Li-hong Yang, Si-lu Wang, Li-li Tang, Biao Liu, Wen-le Ye, Ling-ling Wang, Zhang-yang Wang, Meng-tao Zhou, Bi-cheng Chen

**Affiliations:** 1 Clinical Laboratory Diagnostic Centre, The First Affiliated Hospital, Wenzhou Medical University, Wenzhou, Zhejiang, 325000, Peoples Republic of China; 2 Wenzhou Key Laboratory of Surgery, Department of Surgery, The First Affiliated Hospital, Wenzhou Medical University, Wenzhou, Zhejiang, 325000, Peoples Republic of China; Deutsches Krebsforschungszentrum, Germany

## Abstract

RT-qPCR is the accepted technique for the quantification of microRNA (miR) expression: however, stem-loop RT-PCR, the most frequently used method for quantification of miRs, is time- and reagent-consuming as well as inconvenient for scanning. We established a new method called ‘universal stem-loop primer’ (USLP) with 8 random nucleotides instead of a specific sequence at the 3′ end of the traditional stem-loop primer (TSLP), for screening miR profile and to semi-quantify expression of miRs. Peripheral blood samples were cultured with phytohaemagglutinin (PHA), and then 87 candidate miRs were scanned in cultured T cells. By USLP, our study revealed that the expression of miR-150-5p (miR-150) decreased nearly 10-fold, and miR-155-5p (miR-155) increased more than 7-fold after treated with PHA. The results of the dissociation curve and gel electrophoresis showed that the PCR production of the USLP and TSLP were specificity. The USLP method has high precision because of its low ICV (ICV<2.5%). The sensitivity of the USLP is up to 10^3^ copies/µl miR. As compared with the TSLP, USLP saved 75% the cost of primers and 60% of the test time. The USLP method is a simple, rapid, precise, sensitive, and cost-effective approach that is suitable for screening miR profiles.

## Introduction

MicroRNAs (miRs) are endogenous small interfering RNA molecules regarded as major regulators in eukaryotic gene expression (averaging 22 nucleotides, ranging from approximately 18 to 25 nucleotides in length). These non-coding functional RNA, many of them are phylogenetically conserved [1]. miR play important roles in the regulation of target genes by binding to complementary regions of messenger transcripts to repress their translation or regulate degradation. MiR have been reported to be major modulators in several cellular and pathological processes, including organ development, cell death, cell proliferation, haematopoiesis and patterning of the nervous system [2]. To date, over 2000 miRs have been discovered, including 2,578 in humans, 1,908 in mice and 728 in rats [3].

In the literature, many miR detection methods have been presented: Northern blotting, it needs high RNA concentrations and the method’s drawback is low-throughput[4]; in situ hybridization, this method’s throughput is low as well[5]; small RNA library sequencing, high fabrication rates is disadvantage of the technique [6]; bead arrays, it requires both PCR and hybridization[7]. As well as small library sequencing the drawback to microarray hybridization is high fabrication rates[8]; and reverse transcription PCR [9]. In 2005, Chen et al. first proposed a novel real-time quantification method for the reliable and sensitive detection of mature miRs. Due to high accuracy and sensitivity, stem-loop qRT-PCR became a popular miR detection method in the biomedical field; however, this method needs primers to be designed for each specific miR to make a reverse transcription (RT) and PCR, in other words, specific stem-loop primer is needed for each one miR [9]. In order to develop a new method that can be used for screening and quantifying miR, as well as saving time and reagent costs. We improve upon the TSLP method, and optimize the experimental methods to establish a PCR assay for screening and quantification of miRs, and evaluate its detection ability.

## Material and Methods

### Patients and control samples

During the study period from September 2012 to April 2013, eleven healthy persons and 14 immuno-suppressed kidney transplant recipients were enrolled from The First Affiliated Hospital of Wenzhou Medical University. A sample of 3 ml of venous blood was extracted from each subject, anti-coagulated with heparin, and prepared for cell culture and miR analysis. The surveyed recipients were aged from 25 to 68 years old, and the average age was 48.73 years (12 male and 2 females), received tacrolimus-based immunosuppressive therapy with steroids and mycophenolate mofetil. Healthy persons were aged from 29 to 49 years old, and the average age was 37.57 years (9 male and 2 females). This study was approved by ethics committee of The First Affiliated Hospital of Wenzhou Medical University, approval number: CR201303. Written informed consent was obtained from all participants. In a case of possible compromised capacity to consent, written informed consent was obtained also from a close relative of the participant. This study was conducted according to the principles expressed in the Declaration of Helsinki.

### Reagents and Instruments

Whole blood culture medium (Baidi, Guangzhou, China), T lymphocyte isolation Kit (One Lambda, California, USA), magnetic bead separator (One Lambda, USA), TRIzol Reagent (P/N: 15596-026, Ambion, California, USA), RevertAid First Strand cDNA Synthesis Kit (Thermo, MA 02454, USA), SYBR green fluorescence quantitative PCR reagent kit (Toyobo, Osaka Japan), CO_2_ incubator (Thermo, MA 02454, USA), 7500 real-time quantitative PCR instrument (Applied Biosystems, California, USA).

### Selection and acquisition immune-related miRs

A search was performed in the PubMed literature database, identifying 87 miRs that may affect the function of immune cells: miR-1-3p, miR-9-5p, miR-21-5p, miR-22-3P, miR-26(26a-5p, 26b-5p), miR-27 (27a-3p, 27b-3p), miR-28-5p, miR-31-5p, miR-34a-5p, miR-92(92a-3p, 92b-3p), miR-96-5p, miR-101-3p, miR-103a-3p, miR-106 (106a-5p, 106b-5p), miR-125(125a-3P, 125b-5p), miR-126-3p, miR-142(142-5p, 142-3p), miR-143-3p, miR-146(146a-5p, 146b-5p, 146b-3p), miR-148(148a-3p, 148b-3p), miR-150-5p, miR-152-3p, miR-153-3p, miR-155-5p, miR-181(181a-5p, 181b-5p, 181c-5p, 181d-5p), miR-182-5p, miR-184, miR-187-3p, miR-191-5p, miR-192-5p, miR-193a-3p, miR-193b-3p, miR-194-5p, miR-196a-5p, miR-196b-5p, miR-205-5p, miR-211-5p, miR-221-3p,, miR-296-5p, miR-296-3p, miR-301a-3p,miR-326, miR-339-3p, miR-342-3p, miR-365a-3p, miR-382-5p, miR-424-5p, miR-452-5p, miR-494-3p, miR-498, miR-500(500a-5p, 500b-5p), miR-510-5p, miR-17∼92 (miR-17-5p, miR-18a-5p, miR-19a-3p, miR-20a, miR19b-3p), miR-106a∼363(miR-106a, miR-18b, miR-20b, miR-363-3P), The miR-15 family(miR-15a-5p,miR-15b-5p, miR-16-5p, miR-497), The miR-200 family(miR-200a-3p, miR-200b, 200c-3p, miR-429), Let-7 family (Let-7a,Let-7f, Let-7g, Let-7i, Let-7d).

Sequences of mature miRs were obtained from the database (http://www.mirbase.org/) [3].

#### Design of a universal stem-loop qRT-PCR method for quantification of mature miRs

**Figure 1:**
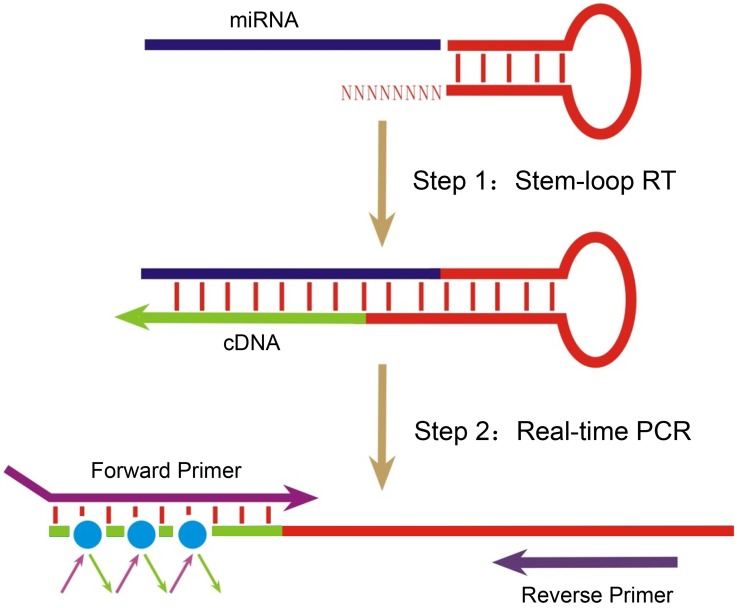
Diagram of USLP method on screening miRs. Light blue lines indicate total RNA. Arrowheads indicate directions of polymerization. 8 random sequences at the 3′ end of stem-loop primer (red) can bind to all mature miRs. In the reaction, mature miR was reversely transcribed into cDNA under the reaction conditions of RT; then, miR was screened and quantified by fluorescence quantitative PCR using miR specific forward primer and universal reverse primer. The forward PCR primer adds additional length with nucleotides that optimize its melting temperature (Tm) and enhance assay specificity. A universal reverse primer that is complementary to a sequence within the RT stem-loop primer.

We proposed a new method based on universal stem-loop qRT-PCR for miR profile screening and quantification [9] (Fig. 1). In order to avoid non-specific amplification, the sequence of the stem and loop is a fragment of DNA from rice genome. The 3′ end tail – USLP has a tail of 8 random tail nucleotides to combine with all mature miR instead of 8 specific nucleotides which complementing specific mature miR sequence. Stem sequence has more binding strength than 8 random sequence for avoid form 3′end mis-match. Moreover, by using human Nucleotide BLAST program, no sequence which is significant similar with the USLP was found.

### Primers in detail and miR-155 standard

1. *Universal stem-loop primer:* 5′-GAAAGAAGGCGAGGAGCAGATCGAGGAAGAAGACGGAAGAATGTGCGTCTCGCCTTCTTTCNNNNNNNN-3′. This sequence was selected from the rice genome and modified using Primer Premier 5. The underlined Ns are random nucleotides.
2. *miR-150 stem-loop primer:* 5′-GAAAGAAGGCGAGGAGCAGATCGAGGAAGAAGACGGAAGAATGTGCGTCTCGCCTTCTTTCCACTGGTA-3′. The underlined nucleotides are complemented with miR-150.
3. *miR-155 stem-loop primer:* 5′-GAAAGAAGGCGAGGAGCAGATCGAGGAAGAAGACGGAAGAATGTGCGTCTCGCCTTCTTTCACCCCTAT-3. The underlined nucleotides are complemented with miR-155.
4. *Forward qPCR primer for miR150:* 5′-GCTCTCCCAACCCTTGTACC-3′. The purpose of the 2 additional 5′nt that are underlined is to get the Tm to 58.5°C, being close to the reverse primer’s Tm (57.9°C).
5. *Forward qPCR primer for miR155:* 5′-GCGGTTAATGCTAATCGTGATA-3′. The purpose of the 4 additional 5′nt that are underlined is to get the Tm to 57.9°C, being close to the reverse primer’s Tm (57.9°C).
6. *Reverse qPCR Primer:* 5′-CGAGGAAGAAGACGGAAGAAT-3′. After reverse transcription of all miRs to cDNA by the same 61 nts USLP, a universal PCR primer is derived from sequences within USLP.
7. *Synthetic miR-155 standard:* 5′- UUAAUGCUAAUCGUGAUAGGGGU-3′. This fragment is used to validate our new method. All primers and synthetic miR-155 standard were purchased (YingJun Biotechnology Co., Ltd., Shanghai, China).
8. *U6 for qRT-PCR:* RT primer: CGCTTCACGAATTTGCGTGTCAB; Forward qPCR primer: GCTTCGGCAGCACATATACTAAAAT; Reverse qPCR Primer: CGCTTCACGAATTTGCGTGTCAT. We chose the small nuclear RNA (snRNA) U6 as a housekeeping gene.

### Peripheral blood cell preparation

Two (2) ml peripheral blood was extracted and anticoagulated with heparin, and 0.5 ml whole blood was added into 5 ml cell culture medium (Baidi, Guangzhou, China) containing 1640 medium, 20% serum, 5 mg PHA, and pH7.2 then cultured for 0, 24, 48, and 72 h at 37°C and 5% CO_2_ humidified incubator. The supernatant was clear, considered without grow of bacterium.

### T lymphocyte isolation and RNA extraction

The cultured cells were centrifuged at 500×g for 5 min, and 1 ml supernatant was left in a 5 ml tube. Added to this were 30 µl anti-CD3 beads that had been resuspended thoroughly before use (vortexed approximately 10 seconds). The tube was immediately capped and inverted 2–3 times to disperse magnetic beads. The tube was then rotated once per second for 3 minutes at 20–25°C to allow binding of beads to T cells. Tube was then uncapped and placed in the magnetic separator for a full 3 minutes. Supernatant was removed and discarded with a disposable pipette, and then the tube was removed from the magnet. Cells (beads) were resuspended with 1–2 ml PBS. The tube was gently flicked to disperse beads and then was replaced in magnetic separator for 1 min. Supernatant was removed and discarded. This process was repeated twice. Total RNA was extracted using TRIzol Reagent (P/N: 15596-026, Ambion, California, USA) according to the manufacturer’s instructions.

### Reverse transcription (RT) and Real-time PCR

Reverse transcription was performed using the RevertAid First Strand cDNA Synthesis Kit (Thermo Scientific, USA) as per the manufacturer’s instructions. The RT reaction was performed using treated total RNA and the RT primer TSLP or USLP. The 10 µl of RT reaction mixture contained 1 µl of treated RNA (0.1 ng–5 µg), 1 µl of RT primer (5 µM) and 1 µl of U6 RT primer (5 µM), 1 µl of 10 mM dNTP Mix, 2 µl of reaction buffer, 0.5 µl of Ribolock RNase inhibitor (20 U/µl), 0.5 µl revertAid M-MuLV Reverse Transcriptase (200 u/µl). The mixture was incubated at 25°C for 5 min, and then incubation was continued at 42°C for 60 min. The reaction was inactivated by heating at 70°C for 5 min. The RT reaction was performed in triplicate to remove RT outliers.

Real-time PCR was performed using SYBR green fluorescence quantitative PCR reagent kit (Toyobo, Code No. QPS-201T) on a 7500 real-time quantitative PCR instrument (ABI, USA), and each sample was analyzed in triplicate. The 10 µl PCR volume included 1 µl of RT product, 5 µl of SYBR Green real-time PCR Master Mix, and 1 µl of primer (forward and reverse, 1 µM each). The reactions were incubated at 95°C for 3 min, followed by 40 cycles of 95°C for 5 s, 62°C for 35 s.

The level of miR expression was measured using the Cq (quantification cycle) value. A synthetic miR-155 molecule was used to calculate the standard curve. MiR expression assay was used and quantified by the comparative 2^−△△Cq^ method and normalized to U6 expression [10], [11].

We chose three evaluation criteria to estimate the sensitivity, specificity and precision of the USLP method. Sensitivity: A series of quantified synthetic miR-155 (10^3^–10^9^ copies/µl) are applied to analyze the dynamic range of the approach. Specificity: The dissociation curve and gel electrophoresis of the qPCR products using USLP method for verifying the specificity of the method. Precision: Intra-assay Coefficient of Variation (ICV) with detecting standard miR-155 is repeated for 20 times to assess the precision of the USLP method.

### Statistical analysis

SPSS12.0 software was used for statistical analysis. Cq values of repeated experiments were expressed as x±s. Cq values detected by the two methods in the sensitivity analysis were analyzed by paired samples T test; the Cq values of miR-150 and miR-155 relative quantification before and after T lymphocyte activation showed skewed distribution, and were analyzed by non-parametric tests (Mann-Whitney *U*-test, for un-normality of the data). Correlation analysis and linear regression analysis were used for analyzing the correlation of the two methods. P<0.05 was considered statistically significant.

## Results

### Optimization concentration of USLP for miR detection

**Figure 2:**
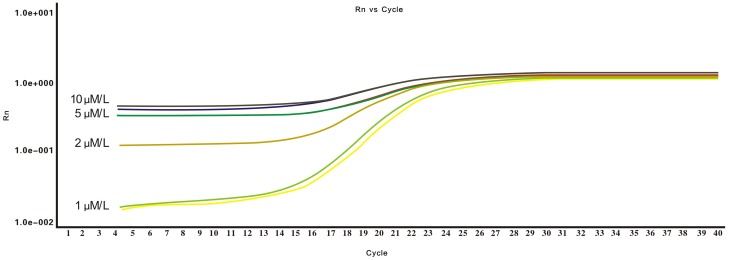
Optimization of qPCR for USLP concentration. USLP at the concentration of 1 µmol/L had higher detection efficiency than others.

When the primer concentrations (10∼1 µmol/L) reached 1 µmol/L in the reverse transcription using USLP, the PCR baseline decreased to a minimum of 10^−2^, and the Cq values appeared two cycles earlier (Fig. 2). This indicates that optimizing the concentration of USLP can cause an earlier rise of fluorescence value, an increased curve slope and improved amplification efficiency. We conclude that the reaction concentration of 1 µmol/L had higher detection efficiency for PCR. According to melting curve analysis, the optimal range of amplification temperature was from 62°C to 65°C.

### Methodology evaluation of USLP method

Synthetic miR-155 standard was used in evaluating USLP method – including sensitivity analysis, specificity analysis and precision analysis – according to protocols as presented above.

**Figure 3:**
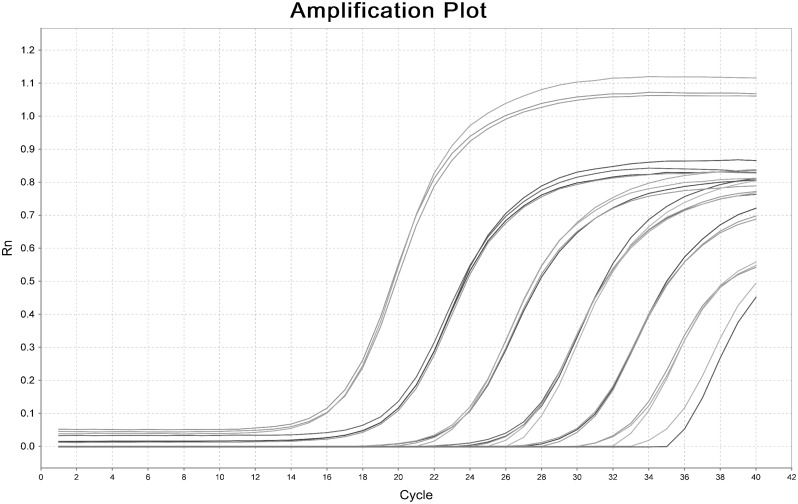
Dynamic range and sensitivity analysis of USLP for measurement of synthetic miR-155. Synthetic miR-155 was diluted by more than seven orders of magnitude from 10^3^ copies/µl to 10^9^ copies/µl.

**Figure 4:**
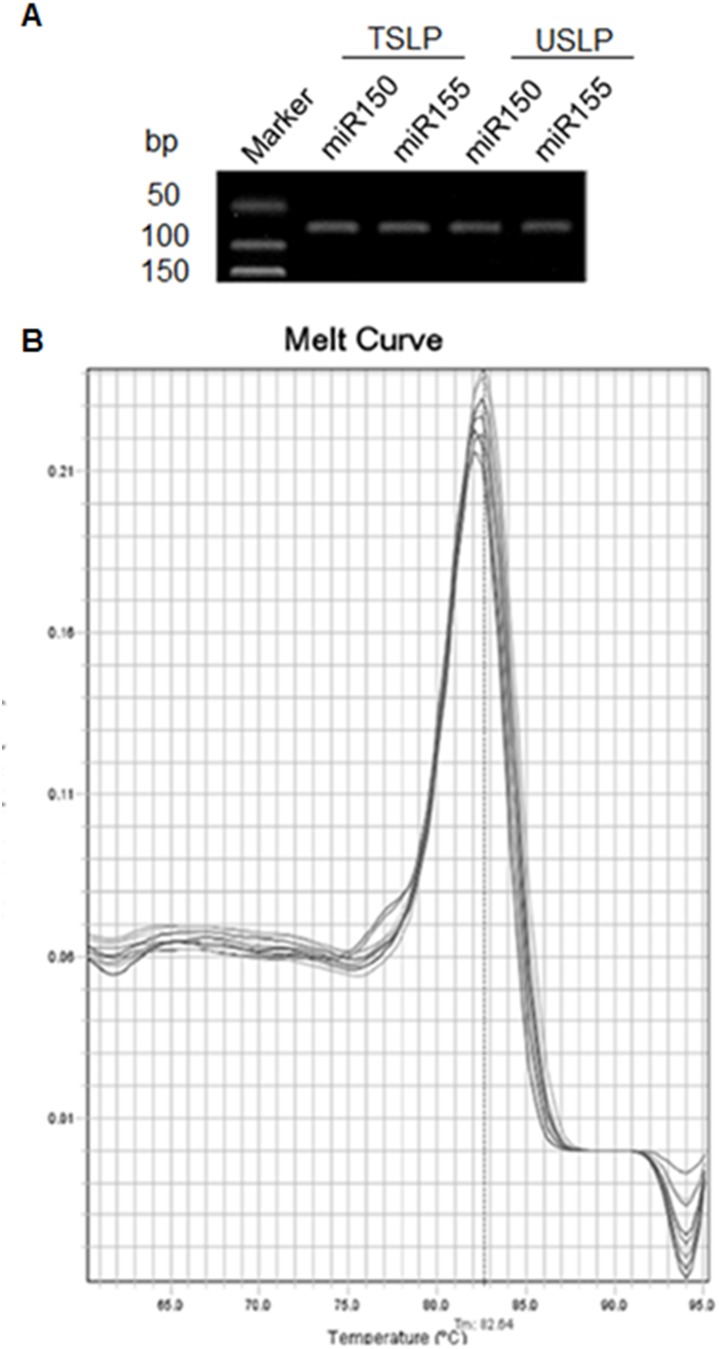
Specificity evaluation of the TSLP and USLP. (A) PCR bands of miR-150 and miR-155 were detected by both TSLP and USLP methods. The PCR products were run on 3% agarose gel in 1X TBE stained with ethidium bromide. (B) Melting curve of the TSLP and USLP methods for detecting synthetic miR-155.

1. *Sensitivity analysis*. Synthetic miR-155 standard was diluted by seven orders of magnitude (final concentration 10^3^∼10^9^ copies/µl), and USLP method assay in Fig. 3 demonstrated that the assay is capable of detecting as few as 10^3^ copies in the PCR reaction.
2. *Analysis of specificity.* The correct PCR product sizes of 64–67 bp were verified by gel electrophoresis (Fig. 4A), while the dissociation curve showing a unique peak from the PCR amplification of synthetic miR-155 in USLP and TSLP, which were superimposed together and attested to the specificity of the amplification.(Fig. 4B).
3. *Analysis of precision.* Under the optimized reaction conditions, Cq values of miR-155 detection ranging from 10^8^ to 10^6^ copies/µl were 19.05±0.29, 22.72±0.09, and 26.54±0.34, respectively, the CV of which were 2.10%, 0.70% and 2.10% (Table 1). The results showed that improved USLP method for miR-155 quantification had high precision and 107 copies/µl was recommend.

**Table 1:** The detection of different concentrations of miR-155 in USLP method.

Standard concentration(copies/µl)	number of detections	Ct (x±s)	CV (%)
10^6^	20	26.54±0.35	2.10
10^7^	20	22.72±0.09	0.70
10^8^	20	19.05±0.29	2.10

### Correlation of the two methods

**Figure 5:**
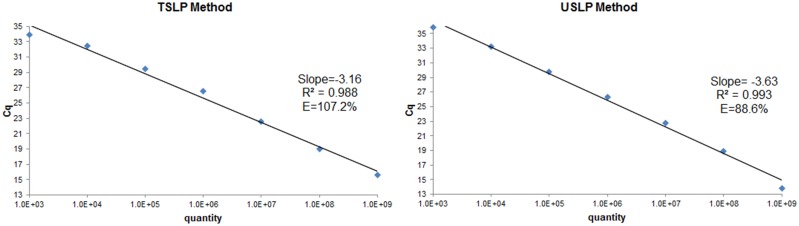
Standard curve of the TSLP and USLP methods for measurement of miR-155. Curve of the new method assay was a straight line (R^2^ = 0.993) with a slope of 3.63 (PCR efficiency = 88.6%) over seven orders of magnitude of Synthetic miR-155. Curve of TSLP method was also a straight line (R^2^ = 0.988) with slope of 3.16 (PCR efficiency = 107.2%) over seven orders of magnitude of Synthetic miR-155.

1. *Amplification efficiency*. After seven gradient dilution, amplification efficiency of the new approach (88.6%) was comparable to the TSLP assay (107.2%), and correlation coefficients (R^2^) of both methods were greater than 0.98 (Fig. 5).

**Figure 6:**
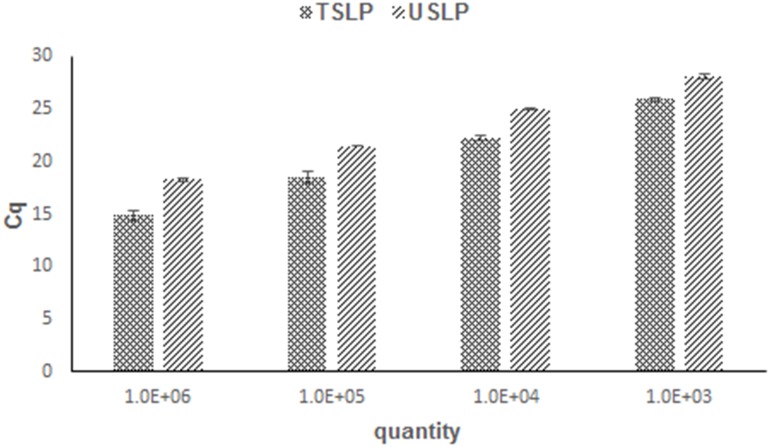
Synthetic miR-155 was diluted by over four orders of magnitude and qRT-PCR using the TSLP and USLP methods.

An interesting phenomenon found in Fig. 6 is that the Cq values of the TSLP method are always lower than USLP in the dynamic range of 2.2–3.4. We speculate that this may be due to lower concentration of cDNA being synthesized by USLP with 8 random nts primer.

1. *Times and costs*. The improved USLP method only needs one kind of stem-loop primer for all mature miRs in the reverse transcription reaction, but the TSLP method needs specific stem-loop primers each under the same conditions. This caused decreased primers and labor consumption. By comparison with TSLP, the USLP method has advantages in time consumption (85 vs. 205 min, saving 60% testing time) and reagent consumption (1917 vs. 7851 bp, saving 75% cost of primers).

### miR expression profile of 87 miRs in T lymphocyte

**Figure 7:**
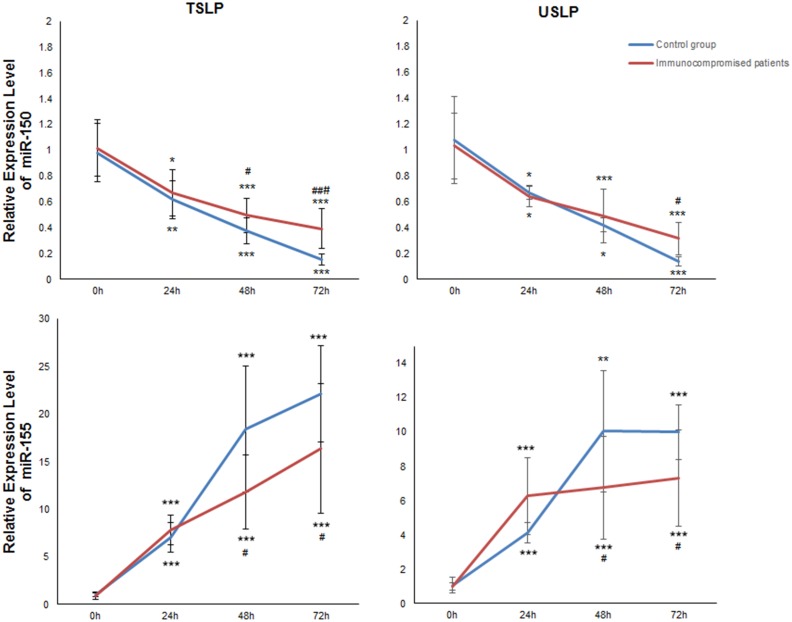
Expression levels of miR-150 and miR-155 in T lymphocytes by using the TSLP and USLP. Expression of miR-150 was decreased nearly 10-fold with prolonged incubation time (incubated at 0, 24, 48, and 72 h). Meanwhile, the expression of miR-155 was increased more than 7-fold with prolonged incubation time. The results of the TSLP and USLP methods were basically consistent in the expression of miR-150 and miR-155. *P<0.05, **P<0.01 and ***P<0.001 vs 0 h; ^#^ *P<*0.05, ^##^ *P<*0.01 and ^###^ *P<*0.001 vs control group.

Optimization of the proposed miR quantification technique was required for practical applications. For experimental validation of the assay, it needed to be validated with biological samples. We detected the expression of 87 miRs in T lymphocytes that were stimulated by PHA, the expression of miR-150 and miR-155 we found were changed significantly. After measuring the expression of the small nuclear RNA (snRNA) U6 as a housekeeping gene, the miR data were normalized by calculating the relative 2−△△Cq value. The expression level of miR-155 was enhanced more than 7-fold as shown in Fig. 7 (P<0.001) while miR-150 decreased nearly 10-fold (P<0.001) over time. Begin with 48 h, healthy persons had lower expression of miR-150 and higher expression of miR-155 compared to the patients. By comparison, USLP couldn’t detected the expression change of miR-150 at 48 h to the patients had statistics significantly.

## Discussion

Since the discovery of the first miRNA in 1993, a surge of interest in miRNA characterization has facilitated the development of highly sensitive and specific miRNA detection techniques to identify disease-related miRNAs and their regulation mechanism. Current methods for miRNA quantification are largely based on microarrays and real-time PCR (stem-loop qRT-PCR especially), which are time-consuming and expensive [12]. Thus, rapid and simple methods for microRNA detection are needed. Here, we provide an improved method for making the study of miR faster and more cost-effective.

Stem-loop RT primers we found to have better specificity and sensitivity than conventional linear RT primers. Secondly, the spatial constraint of the stem-loop structure might prevent the RT primer from binding double-stranded genomic DNA molecules, and enhance the thermal stability of the RNA-DNA heteroduplex [13]. Therefore, we adopt the stem-loop method as the basis for the design of the screening and quantitative method. The USLP method was evaluated with high sensitivity, specificity and precision.

First of all, compared with USLP method, the TSLP method has certain advantages of high sensitivity; however, it requires that specific stem-loop primers (70∼80 bp) be designed for each miRs, which is time-consuming and costly. With the USLP method, though, reverse transcription of 87 miRs can be finished in one tube. Secondly, microarray is high-throughput, whereas the methods are expensive for screening all of miRs in specimen. The USLP method also can determine a number of miRs and cheaper than microarray. Thus, this method makes it simple to screen massive miRs and find miR biomarkers of certain diseases. In addition, it improves the use efficiency of the specimens, and reduces the number of required specimens.

However, by compared with TSLP, the sensitivity of the USLP method was lower and would not be able to detect and provide an accurate quantification of low expressing miRs for the majority of miRs. (Figs. 5–6). We speculate that this may be due to lower concentration of cDNA being synthesized by USLP with 8 random nts primer. Therefore, the USLP method is more suitable for screening massive miRs rather than quantifying low level expression of particular miRs.

In this study, we screened 87 miRs by USLP, and found that the expression of miR-150 and miR-155 undergoes significant changes during T lymphocyte activation. Furthermore, miR-150 functions in hematopoiesis; it regulates genes whose downstream products encourage differentiating stem cells to become megakaryocytes rather than erythrocytes. It is also thought to control B and T cell differentiation, alongside mir-155 [14], [15]. Rodriguez et al found that miR-155 may play roles in regulating differentiation of T lymphocyte towards type 1 helper T lymphocytes (Th1), and stimulating type 2 helper T lymphocytes (Th2) to secrete interleukin 4 (IL-4), IL-5 and IL-10 [16]. The data clearly demonstrated that the contents of miR-150 and miR-155 are associated with the activation of T lymphocytes, which may be meaningful to the assessment of individual immune status.

For the sake of discoveries disease- or tissue-specific miR biomarkers, the method that can screen whole miR expression profile is urgently required. Our data showed that the USLP method combined with the quantitative PCR technique was fast, stable, sensitive, time-saving and economic for miR profile screening and miR quantification. Using the improved USLP method can enhance the screening efficiency of miRs by qRT-PCR, and provide a new technology platform for miR research.
